# Dr. Eugene F. Hoyt

**Published:** 1913-08

**Authors:** 


Dr. Eugene F. Hoyt, Hahnemann of New York, 1870, a practitioner of New York City, died in Newark, N. J., early in June. He was born in Niagara County in 1846.

